# Effect of Intra-Articular Hyaluronic Injection on Postural Stability and Risk of Fall in Patients with Bilateral Knee Osteoarthritis

**DOI:** 10.1155/2014/815184

**Published:** 2014-06-19

**Authors:** Nafiseh Khalaj, Noor Azuan Abu Osman, Abdul Halim Mokhtar, John George, Wan Abu Bakar Wan Abas

**Affiliations:** ^1^Department of Biomedical Engineering, Faculty of Engineering, University of Malaya, 50603 Kuala Lumpur, Malaysia; ^2^Department of Sport Medicine, Faculty of Medicine, University of Malaya, 50603 Kuala Lumpur, Malaysia; ^3^Department of Biomedical Imaging, Faculty of Medicine, University of Malaya, 50603 Kuala Lumpur, Malaysia

## Abstract

Knee osteoarthritis is a common cause of disability which influences the quality of life. It is associated with impaired knee joint proprioception, which affects postural stability. Postural stability is critical for mobility and physical activities. Different types of treatment including nonsurgical and surgical are used for knee osteoarthritis. Hyaluronic acid injection is a nonsurgical popular treatment used worldwide. The aim of this study was to demonstrate the effect of hyaluronic acid injections on postural stability in individuals with bilateral knee osteoarthritis. Fifty patients aged between 50 and 70 years with mild and moderate bilateral knee osteoarthritis participated in our study. They were categorized into treatment (*n* = 25) and control (*n* = 25) groups. The treatment group received five weekly hyaluronic acid injections for both knees, whereas the control group did not receive any treatment. Postural stability and fall risk were assessed using the Biodex Stability System and clinical “Timed Up and Go” test. All the participants completed the study. The treatment group showed significant decrease in postural stability and fall risk scores after five hyaluronic acid injections. In contrast, the control group showed significant increase. This study illustrated that five intra-articular hyaluronic acid injections could significantly improve postural stability and fall risk in bilateral knee osteoarthritis patients. This trial is registered with: NCT02063373.

## 1. Introduction

Knee osteoarthritis (OA) is a common cause of disability, particularly among elderly population [1, 2]. Knee OA is associated with pain and progressive loss of mobility and function [3, 4] including gait, stair climbing, and other physical activities which involve lower limbs. In addition, people with knee OA experience loss of proprioception [5–8], which may affect postural stability (static and dynamic balance) and risk of fall [9]. Postural stability could be defined as control over body's position in space for orientation and balance purpose [10]. Maintaining postural stability is essential for us to maintain postural stability during activities of daily living (ADLs) and ambulation. Impaired postural stability is one of the main reasons of falls in older adults and thus constitutes a significant public health problem [11]. It is considered as one of the leading causes of fatalities and hospital admissions [12].

Current treatments for knee OA are analgesics or nonsteroidal anti-inflammatory drugs (NSAIDs), surgical treatments (i.e., arthroscopy and total knee replacement), intra-articular injections, physiotherapy, weight reduction, exercise, orthotic (braces), and patient education [1, 13]. The majority of treatments are nonoperative that help to improve pain and function. Intra-articular hyaluronic acid (HA) is one of the nonoperative treatments used to improve pain and articular function in knee OA [1, 13].

HA is a major component of both synovial fluid and articular cartilage and is responsible for the elastoviscosity of synovial fluid that allows efficient movement of articular joint [2, 14, 15]. The quantity of HA (the concentration and molecular weight) in the synovial fluid is decreased in osteoarthritic knee [13, 16, 17], which would expose the knee to potential physical damage and limit its role in maintaining normal joint biomechanics [3, 13, 17]. HA injections are used for the purpose of viscosupplementation by replacing the lost HA and stimulating the production of endogenous HA within the osteoarthritic joint [17, 18]. HA applies anti-inflammatory, analgesic, and probably chondroprotective effects on the cartilage and joint synovium [18]. HA has been used for treatment of mild and moderate knee OA for a long time and was proven that they are effective in reducing pain perception [19].

Studies that evaluate postural stability and fall risk in knee OA patients after receiving intra-articular HA injections are very limited. According to our knowledge, only one study to date has assessed the effect of HA on balance in geriatrics with knee OA using clinical tests [1]. This study is the first to determine the effect of HA injections on postural stability and fall risk using the Biodex Balance System (BBS) and clinical “Timed Up and Go” test (TUG). The purpose of this study was to demonstrate the effect of five intra-articular HA injections on postural stability and risk of fall in individuals with bilateral knee OA.

## 2. Methods

### 2.1. Ethical Statement

The Medical Ethics Committee in the University Malaya Medical Centre (UMMC) (919.18/May 2012) approved this study. All participants read and signed a written consent form.

### 2.2. Participants

Fifty subjects with bilateral mild and moderate knee OA voluntarily participated in this study and were categorized into treatment and control groups. They were referred to from UMMC, Kuala Lumpur, Malaysia. All participants underwent clinical assessment, which was conducted by a medical doctor. Diagnosis of knee OA was confirmed by knee X-ray (anterior-posterior and medial-lateral views). Two specialists (radiology and sports medicine) graded knee X-rays using Kellgren-Lawrence grading scale.

Participants with knee OA were included if their age was between 50 and 70 years, had bilateral knee OA grades II (mild) and III (moderate), and were independent of ADLs. Participants were excluded if they had any lower limb deformities (for instance, flexion contracture of the knee joint, knee hyperextension, knee valgus, and varus and hallux valgus), lower limb joint replacement, knee surgery for the past 12 months, any lower limb fractures during the past six months, intra-articular injection in the previous 6 months, neurological disorders, diabetes mellitus, history of recent fall (past 12 months), and having underwent any treatment and rehabilitation program.

The participants were classified into the following two groups: treatment group (received intra-articular HA injections) and control group (no treatment). Anthropometric data of all participants were obtained before the assessment of balance and risk of fall. All the participants underwent two assessment sessions which were before and after the injections for the treatment group and weeks one and six in the control group. Measurements and injections were done by two different researchers to avoid bias.

### 2.3. Treatment Procedure

All the participants were asked to stop taking medicine or supplements that were taken for their knees, ranging from painkillers to glucosamine. In addition, during this study no treatments were permitted for their knees. Twenty-five individuals (50 knees) with mild and moderate knee OA underwent five weekly intra-articular injections of HA-Hyalgan 20 mg/2 mL (Fidia, S.p.A, Abano terme, Italy) into both knees. They were asked to report any adverse events that they see and feel after the injections. HA has been shown to be safe [3, 14]; however, common adverse events of HA injections are inflammatory reaction at injection site, itching, headache, and calf pain [13]. In this study no adverse events were observed in the participants.

### 2.4. Protocol of Postural Stability and Risk of Fall Assessment

Balance and risk of fall were assessed using the Biodex Stability System and TUG test. The TUG is an internationally accepted functional, dynamic test of balance with known reliability and validity; this test is low cost and easy to apply [20]. The TUG test measures the time in seconds that takes a subject to stand up from a chair, walk three meters at a comfortable and safe pace, turn around, walk back to the chair, and sit down [20]. Subjects with scores <10 s, <15 s, <20 s, and >30 s are considered normal, at risk of falling, able to walk and climb the stairs independently, and unable to sit and climb the stairs without help, respectively [20]. In this study, participants were asked to perform TUG three times.

The BSS (Biodex Medical System Inc., Shirley, NY, USA) is a commercial balance device which was designed to assess and record balance and neuromuscular control under dynamic stress [9]. The BSS is multiaxial device with an unstable balance platform, which provides up to 20° surface tilt in a 360° range of motion, to measure postural stability under dynamic tests. This platform is free to move about the anterior-posterior (AP) and medial-lateral (ML) axes simultaneously [21], thereby permitting the acquisition of three measurements, namely, overall stability index (OSI), AP stability index (APSI), and ML stability index (MLSI) [9]. BSS provides 12 levels for assessing balance and risk of fall, in which level 12 is the most stable and level 1 is the most unstable (difficult task). The OSI is considered as the most reliable indicator of postural stability. Arnold and Schmitz (1998) suggested that the overall score is the tool that can be used to assess balance [21]. Overall score can be affected by AP and ML scores. Low OSI indicates better balance, and high score indicates poor balance. In addition, the risk of fall can only be presented by the actual score in the OSI.

Postural stability and risk of fall of all participants were assessed using BSS. The participants were asked to step on the platform in a bipedal stance with bare feet and open eyes [11] looking forward to the monitor (BSS monitor), while their hands are hanging by their sides (hand support was not permitted). They were asked to stand straight, not to change their feet position, and only sway their body when it was needed. Handles were available for safety purpose, but touching the handles would cancel the trial.

Failure to control the positioning of the feet may significantly confound explanation of clinical or experimental balance measures [22]; in other words, foot placement can affect stabilizing reactions. However, positioning the feet outside of the subject's or patient's preferred (comfortable) stance position may affect the measured postural response [22]. Thus, measurements from two different feet positions were used to avoid any bias to the results which were defined and functional positions. The defined position was adopted according to the findings of McIlroy and Maki [22] and was almost the same for all the participants. The defined position was based on the average absolute stance width for elderly at 0.16 (0.04) m (10.4% height) and average stance angle at 16.6° (11.3) [22]. The functional position is the position assumed by the subjects on functional or comfortable standing position [23, 24]. In other words, functional position is the standing position that participants have during walking and other ADLS.

Heel width is defined as the distance between the midlines of the two heels [22]. The feet angle was calculated between the lines joining the centre of the heel and the great toe of each foot [22]. Foot angle and heel width in the defined position was 20° and 17.6 cm average, respectively. In the functional position, the average and SD of these values were 36.65° ± 9.03 and 14.98 ± 3.7 cm.

Postural stability and risk of fall were assessed with two trials over a period of 30 seconds with 10 seconds rest in between. Participants were given 5 minutes rest between two testing positions. The order of testing was random. Assessing dynamic bilateral stance was conducted on the setting platform at level 8 [23]; for static level, the platform was set to remain static with no tilt. Prior to the testing, participants underwent a familiarization session. The test procedure was briefly explained, and participants underwent only one practice trial to familiarise with BSS and to understand what we wanted them to do.

### 2.5. Statistical Analyses

The SPSS Statistics version 17.0 was used for all statistical analysis. Independent sample* t*-test and paired sample* t*-test were used to assess the difference in postural stability and fall risk scores between two groups and within groups in baseline and week 6 (after the treatment), respectively. In addition, descriptive analysis was used to assess mean and standard deviation (SD) of all the variables. The alpha level of 0.05 was defined as statistically significant for all the tests.

## 3. Results

Fifty knee OA patients participated in this study (14 male and 36 female). All participants were aged-matched and the average of age was 58.63 ± 5.62 years (ranged from 50 to 70 years). The averages of weight, height, and BMI of all the participants were 73.19 ± 11.5 Kg, 1.57 ± .009 meters, and 29.76 ± 4.6 kg/m^2^, respectively.  Table 1 presents anthropometric information of all participants in the control and treatment groups.

**Figure 1:**
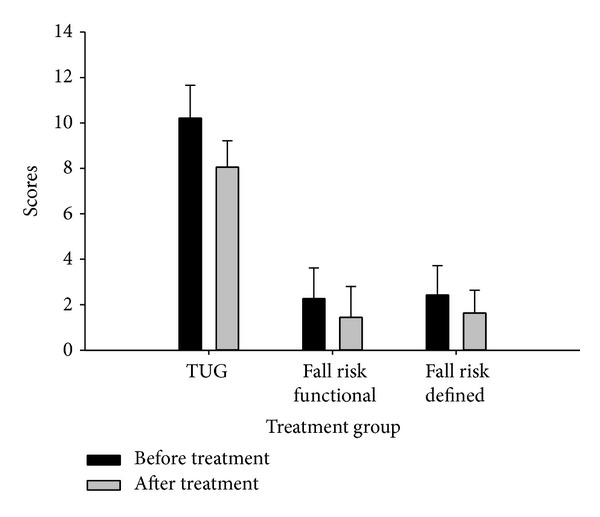
TUG test (in seconds) and fall risk scores before and after treatment.

Table 2 shows the mean and SD results of postural stability scores (static and dynamic balance) of all participants in both functional and defined feet positions. In this table OA, AP, and ML scores are presented in weeks one (before injections) and six (after completing injections).  Figure 1 presents the results of TUG and risk of fall scores in treatment group. The fall risk and TUG scores for control group in weeks one and six were 2.48 ± 1.17, 2.98 ± 1.24, 10.11 ± 1.33, and 10.5 ± 1.38, respectively.

Independent sample* t*-test was used to indicate whether any difference exists in postural stability scores between control and treatment groups in baseline and week 6.  Table 3 presents the results of independent sample* t*-test data. The results showed that there was no significant difference in postural stability scores in baseline; in contrast, there was a significant difference in postural stability scores in week 6.

Paired sample* t*-test was used only for overall scores. The results are presented in Table 4. The results of paired sample* t*-test showed a significant decrease in postural stability, fall risk, and TUG scores in the treatment group after five HA injections. However, static balance-defined feet position did not show any significant difference after treatment. The results of the treatment group confirmed the improvement in postural stability and risk of fall after treatment. Moreover, results showed that there was a significant increase in postural stability, fall risk, and TUG scores in control group, except for static-defined and dynamic-functional feet positions.

## 4. Discussion 

Balance disorders are a growing public health problem due to their association with falls and fall-related injuries. Deficits in lower limb proprioception are associated with knee OA [25, 26]. Decreased postural stability causes difficulties in performing activities of daily living, which would affect the patient's quality of life [11]. Thus, treatments should aim to improve postural stability and decrease the risk of fall in knee OA patients.

According to our knowledge, this study is the first to determine the effect of five HA injections on postural stability and risk of fall in individuals with bilateral knee OA. Finding of this study revealed that there was a significant decrease in postural stability and risk of fall scores in subjects treated with HA in all tests, except for static balance test with defined feet position. To be precise, postural stability improved considerably and risk of fall decreased significantly after five weekly intra-articular HA injections. Generally, postural stability and fall risk in control group worsened during six weeks of this study.

Previous studies assessed the effect of intra-articular HA injections on physical functioning and pain [1, 19, 27]. However, very few studies assessed the effect of HA injections on postural stability in individuals with knee OA. A similar study was done by Sun et al. [1]; they assessed balance of 68 patients with unilateral knee OA after five weekly HA injections using clinical test. They reported a significant improvement in balance and pain after five HA injections [1]. The findings of our study corroborated the results of Sun et al. [1], which show the significant improvement in postural stability of both unilateral and bilateral knee OA patients after receiving five weekly HA injections.

One of the explanatory factors for postural stability impairment and higher risk of fall in knee OA patients is pain. Pain associated with knee OA increased the propensity to trip on an obstacle, and greater pain is associated with a greater risk of fall [28]. These findings underscore the importance of improving pain in individuals with knee OA. HA injection improves pain in osteoarthritic knees [1, 27].

There are varieties of treatment used for knee OA including intra-articular HA injection. HA is a beneficial treatment for knee OA with long lasting symptomatic efficacy and potential positive effects on joint tissues [29]. It was proven that OA progression was less in knee OA patients who received repeated intra-articular HA injection [30]. Moreover, intra-articular HA injection improved the pain in knee OA patients by 20 to 40% over 6 months to a year [7]. HA treatment was effective in improving pain and function, normalising the properties of synovial fluid [2, 15, 31, 32], and accordingly HA improves quality of life in patients with knee OA. Furthermore, HA injection in osteoarthritic knee led to short-term increase in proprioception, isokinetic muscle force, and functional improvement [33]. Consequently, HA intra-articular injections could improve the postural stability and decrease the risk of fall by decreasing pain and improving proprioception.

This study had several limitations. The sample size was small; studies with larger sample size will be beneficial. Severe knee OA patients were not included in this study. The mean of age and BMI of both groups were similar; however, they were not exactly the same. In addition, follow-up studies are needed to evaluate the long-term effect of this treatment.

## 5. Conclusion 

This study aimed to evaluate postural stability and risk of fall in patients with bilateral knee OA who received five weekly HA injections (both knees). BSS and clinical TUG test were used to assess postural stability and risk of fall. We found that patients with bilateral mild and moderate knee OA would benefit from five HA injections in terms of postural stability and fall risk improvement.

Improving postural stability of older adults with knee OA has become an important challenge. Establishing these data has implications in rehabilitation and will enable the practitioner to customize their rehabilitation strategies. Future studies need to determine the long-term effect of HA injections on postural stability and fall risk, as well as including severe knee OA group in their studies. It is also important to determine which severity of knee OA will benefit more from HA injections in order to start the treatment on the appropriate time.

## Figures and Tables

**Table 1:** Anthropometric information of all participants.

	Control	Treatment
	Mean (SD)	Mean (SD)
Age (years)	60.83 (5.9)	56.08 (4.03)
Height (meter)	1.56 (0.09)	1.56 (0.08)
Weight (Kg)	75.44 (9.7)	70.08 (12.42)
BMI (Kg/m^2^)	31.01 (3.3)	28.6 (5.3)

**Table 2:** Postural stability scores.

	Treatment group		Control group	
	Before injections	After injections	First assessment	Second assessment
	Mean (SD)	Mean (SD)	Mean (SD)	Mean (SD)
Static (functional)				
OA	0.7 (0.36)	0.51 (0.16)	0.72 (0.36)	0.84 (0.39)
AP	0.52 (0.31)	0.39 (0.13)	0.53 (0.29)	0.62 (0.32)
ML	0.35 (0.21)	0.26 (0.17)	0.38 (0.26)	0.42 (0.23)
Static (defined)				
OA	0.81 (0.71)	0.55 (0.22)	0.72 (0.30)	0.77 (0.34)
AP	0.64 (0.65)	0.42 (0.19)	0.53 (0.23)	0.56 (0.26)
ML	0.36 (0.28)	0.26 (0.16)	0.37 (0.22)	0.43 (0.23)
Dynamic (functional)				
OA	1.7 (0.80)	0.8 (0.36)	1.49 (0.87)	1.61 (0.76)
AP	1.09 (0.46)	0.56 (0.26)	0.9 (0.56)	1.1 (0.46)
ML	1.07 (0.59)	0.44 (0.25)	0.91 (0.59)	0.94 (0.59)
Dynamic (defined)				
OA	1.75 (0.75)	0.92 (0.31)	1.69 (0.97)	1.82 (0.95)
AP	1.19 (0.42)	0.64 (0.22)	1.07 (0.62)	1.17 (0.49)
ML	1.09 (0.56)	0.52 (0.25)	1.02 (0.63)	1.08 (0.73)

**Table 3:** Independent sample *t* test results.

Tests	*t*	Sig. (2 tailed)
Static defined foot position—baseline assessment	0.22	0.83
Static defined foot position—second assessment	−2.48	0.02^∗^

Static functional foot position—baseline assessment	−1.04	0.31
Static functional foot position—second assessment	−3.52	0.00^∗^

Dynamic defined foot position—baseline assessment	−0.48	0.64
Dynamic defined foot position—second assessment	−4.67	0.00^∗^

Dynamic functional foot position—baseline assessment	−0.02	0.98
Dynamic functional foot position—second assessment	−5.04	0.00^∗^

Risk of fall defined foot position—baseline assessment	−1.22	0.23
Risk of fall defined foot position—second assessment	−4.94	0.00^∗^

Risk of fall functional foot position—baseline assessment	−1.67	0.10
Risk of fall functional foot position—second assessment	−5.63	0.00^∗^

TUG—baseline assessment	−0.13	0.90
TUG—second assessment	−6.05	0.00^∗^

*Significant difference.

**Table 4:** Paired sample *t* test.

	Treatment group		Control group	
	*t*	Sig. (2 tailed)	*t*	Sig. (2 tailed)
Static-defined	1.87	0.074^∗^	−1.93	0.07^∗^
Static-functional	2.86	0.009	−2.67	0.016
Dynamic-defined	7.58	0.000	−3.06	0.007
Dynamic-functional	7.95	0.000	−1.07	0.296^∗^
Fall risk-defined	5.19	0.000	−3.21	0.005
Fall risk-functional	5.18	0.000	−3.56	0.002
TUG	1.16	0.000	−3.62	0.002

*No significant difference.
